# Bursa-Pastoris (Shepherd’s-Purse)

**Published:** 1896-08

**Authors:** J. H. Allen

**Affiliations:** Logansport, Ind.


### BURSA-PASTORIS (SHEPHERD'S-PURSE).

J. H. ALLEN, M. D., LOGANSPORT, IND.

This wonderful remedy, which we find growing by the wayside in most civilized countries and belonging to the natural order of *Cruciferæ*, and known by the common name of Shepherd's-purse, receives but a page and a half in Hering's *Guiding Symptoms.* It has been suddenly brought to our attention by the earnest efforts of Dr. B. Fincke, of Brooklyn, in a very fine proving made by and under his instructions in 1894 and 1895, and published in the International Hahnemannian proceedings of a meeting held at Narragansett Pier in June, 1895. In Hering's *Guiding Symptoms* it is known under the name of Thlaspi-Bursa-Pastoris, but in his proving Dr. Fincke has simplified the name to Bursa-Pastoris. His attention was first called to this remedy by Dr. Anton Hoffman, of Frankfort, Germany, who told him of its wonderful hæmostatic powers. He says a bunch of it held under the arms will stop nose-bleed immediately. His provings are preceded by extracts from old books giving records of the observations of older physicians, and of some cures made by more modern physicians based upon them; also his own proving by the strong tincture, the 30th and 1,000th centesimal potency; also a proving by Dr. Malcolm MacFarlan with the 9,000th potency. The proving of Dr. Fincke has been made with great care to avoid anything that might interfere with the purity of observation. Hippocrates mentions its use in hemorrhages of the uterus, and, according to Plinius, the seeds were used to purify the bile, to promote menstruation, for the removal of internal ulcers and abscesses, and it was known by Discorides to kill the fœtus. It is also mentioned by Paracelsus as curative in dysentery or flux and in menstrual hemorrhages, nose-bleed, and spitting of blood. Many others have mentioned wonderful cures by this remedy, especially in the old school of medicine. The following are the symptoms of

### BURSA-PASTORIS.

MIND.—Great excitement, with red face and ebullition of blood; melancholy and inclined to weep; excitable and angry the whole day (like Nux); lassitude, with disinclination to work (like Sepia); disagreeable, scolding disposition (Sepia); very nervous, irritable, cross; feels like fighting; precise, particular, and exacting; the future looks dark and gloomy; indifferent to life; nervous and excitable, with indifference to life and constant depression of spirits; desperate, discouraged; thinks she must destroy herself, especially when left alone.

SENSORIUM.—Vertigo with sensation as if drunk and with contraction of the brain, as if it was rising out of top of head. Stupefied and dull, as if she had not slept enough. Dizziness, leans on her head with eyes closed, or as if suspended in the air; vertigo worse from rising from a stooping position. Dizziness in the forehead, like sea-sickness, passes off after walking and resting.

HEAD.—Pressing in forehead toward the right side, into right eye, with heaviness in it. (A sensation.) Stupefying, pressing pain in forehead. Headache, with great fullness. Headache beginning in the middle of left eyebrow, running up and over top. Violent headache, lasting all night and worse toward evening; frontal headache worse toward evening.

EYES.—Pressure in forehead toward right side. Sensation as if the ball was pressed inward, with waning of sight in myopia, aggravated by glare of sun. Unpleasant feeling in the morning, as of dust, after sewing or using eyes. Sensation as if swollen.

EARS.—Under this rubric we have many symptoms, but I will give only a few of the most prominent ones: Spasmodic drawing from right ear to right lower jaw. Dry eruptions behind the ears; small vesicles, with yellow points, above right ear. The eruptions come and go frequently. Scaly eruptions behind ears, with fissures in the fold. Sensation as of an insect entering the left ear, making a buzzing noise, and relieved by boring in the ear with fingers.

NOSE.—Gnawing in nasal bones and soreness in nose, with tenderness on touch. Dryness of nose, preceded by sensations of bleeding. Bruised feeling each side of bridge, with bleeding from nose.

FACE.—Face feels parched and dry, miserable countenance, dusky eyes, shiny, and with dilated pupils.

MOUTH AND TONGUE.—Scratchy feeling in the palate. Tongue and palate feel as if scalded. Feel raw, as if from smoking strong tobacco. White-coated tongue, mouth cracked in right corner, and sore. Upper lip cracked.

TEETH.—Drawing in right upper molar and in hollow teeth, worse from cold water (reverse of Pulsatilla), the pain in the teeth is of a dull nature.

THROAT.—Dryness of fauces, with sensation of constriction of the throat, also with dry sensation and scraping. Violent sore throat. All-night chills, with wakefulness and slight diarrhœa, with swelling of tonsils. Sensation as if the throat was laced together.

APPETITE AND TASTE.—Great longing for food, and, while eating, is disgusted with the food, with emptiness in the stomach. Canine hunger; food tastes good, but can eat a very little. Hungry two hours after eating. (Sulph., Ign., Sepia.) Desire for bread and butter, which does not taste good. Aversion to potatoes; vicious appetite, with either constipation or diarrhœa; even water tastes bad, also milk. Oat cakes are eaten with a relish. Bitter, slimy taste, with dryness of mouth and white-coated tongue. Nausea in the forenoon; on walking, nausea with loathing of food. Vomiting and purging, with great debility. Better toward evening and after strawberries; worse after eating. Sour rising, like heartburn.

STOMACH.—All food causes pain and lies heavy on stomach; empty feeling, with bad, bitter taste. Spasm of stomach with nausea; thirst for cold water. Worse after drinking; worse in the forenoon. Sharp pains in lower bowels with an uneasiness in the rectum as of diarrhœa, sometimes with nausea, pains sharp. Bloated after eating, with loud emissions of flatus; bowels feel as if drawn down and out.

STOOL.—Slow and difficult, obstinate slow stool with severe pressure and urging without effect, nine P. M. Hard, difficult, dry stool, leaving anus sore (stool in small balls at times), bleeding after hard stool. Diarrhœa mushy, brown, dark, urgent, and sometimes with nausea, yellowish color, troublesome itching, and protrusion of piles.

BLADDER AND URETHRA.—*Female.*—Heat and irritation in urethra with dryness, surrounding the parts, relieved by application of cold water; urine somewhat scant, urine foamy, light colored, or clayey, and under it a brownish, red deposit.

GENITAL ORGANS.—*Male.*—Stitching in penis, running through to anus. Soreness of prepuce with intense itching or cold sensation (Agar.).

*Female.*—Bearing down in uterine region on rising, relieved by cold water. Weakness in uterine region on rising. Stitch-like pain on right side of uterus, *sore pain in womb on rising,* great weakness on going up-stairs. Weakness in genital organs with pain in back, also accompanied with stiffness in back; pain in lower abdomen with much weakness after dinner, stiffness and weakness in uterine region with the same condition of stiffness in the limbs, worse standing; prostration after pain in uterine region. Bearing-down pressure very low from brim of pelvis, with weakness as before delivery of child, worse on rising in the morning. Pressure in pubic region better by lying down. Pressure in pubic region on rising until eleven A. M. Pressure in vagina in morning, worse on standing, remained until she had lain down two hours, returning after standing, with prostration. Pain in lower back through womb before rising A. M.; pain in womb after coughing or sneezing. Cramping pain across womb on getting out of bed. Weakness in lower abdomen and also in womb, must lie down, better after eating. Since cessation of menses, weakness of womb and pain in back on rising. Weakness in womb relieved by bathing. Weakness of womb at menses, scanty and pale at first, then profuse.

LEUCORRHŒA.—Transparent and followed with prostration. The menses were accompanied with bearing-down pain; scanty and brownish, lasting four days; or bright color and watery, not profuse, with weak feeling in womb, worse from standing. *Coryza*, sneezing, running from nose, cough with hoarseness and oppression of chest, and dull pain over eyes; cough is better in open air.

COUGH.—With irritation behind sternum, as of something tearing away; fits of coughing as if the sternum pressed inward with pain in it when drawing a deep breath; accumulation of phlegm in chest at lowest part of sternum, inducing convulsive cough; relieved as soon as phlegm is loosened; pain, uneasiness, and pressure behind the sternum.

HEART.—Stitching pains at the apex; palpitation worse in the morning in bed; palpitation ending with cough; pain left side of apex, running down to short ribs, region of apex of left heart; sensitive to slight pressure.

BACK.—Morning backache; awakened with violent pain between the shoulders, with great weakness; awakened at four A. M. with violent pain between the shoulders, extending to lower part of body, in sympathy with womb; passed off on rising; pain between shoulders, extending to waist; weakness between shoulders on rising; continued pain in small of back.

UPPER EXTREMITIES.—Strained pain in right shoulder; gouty pain in arm; tearing, gnawing pain from elbow to throat; rheumatic pain in left arm and shoulder-blade, extending to throat; hands tremble when holding anything; swelling of veins of hand and arm; tearing, wrenching pain in left wrist; redness around root of nails; lame feeling in right forefinger; pain in the periosteum.

LOWER EXTREMITIES.—Drawing pain in left thigh like gout; weariness in lower limbs; can hardly stand, with severe pain and stiffness in right knee-cap; tearing, gouty pains left leg, worse in forenoon.

SLEEP AND DREAMS.—Restless tossing about; frequent awakening; ebullition of blood and great heat all over; much fatigue toward evening, with sleeplessness; could not get to sleep before twelve o'clock at night; pain under left shoulder-blade kept her awake; sleepy after eating, or falls asleep while eating; confused and unpleasant dreams when asleep.

CHILL AND FEVER.—Chill at nine A. M., or from seven to nine P. M.; worse from lying down; chilly when uncovering during heat; worse from going into warm room during fever; skin dry, hot, even to burning; continued fever with quick pulse; hot, dry skin; red cheeks; scanty urine; sweat profuse at night; worse after sleep.

GENERALITIES.—Distunement of whole organism; lassitude; disinclination to work; much weakness; can scarcely hold herself up; tremulous; feels like fainting; susceptibility to cold; prostrations; worse toward night; better by cold bathing and open air.

